# The Growth of Animals

**Published:** 1898-06

**Authors:** 


The Growth of Animals.—Different animals, when young, grow with very different degrees of rapidity. Man grows slower than the horse; the horse more slowly than the cow, and the latter slower than the dog. The weight at birth of human babies averages about 3000 grammes in the case of girls, and 3150 grammes in boys. The average weight of a newborn foal is about 50–52 kg.; of a newborn calf, 35.5 kg. A child doubles its weight at birth in about six months; a foal in two months; the calf in six to seven and one-half weeks; a young pig in eighteen days, and a lamb in ten days. The rapidity of growth is therefore very different in different animals.

Prof. von Bunge, in his *Text-book of Chemistry*, advanced the theory some years ago that some relation must exist between the rapidity of growth and the composition of the milk. Fr. Pröscher has lately tried to prove this theory by a series of investigations as to milk made in Bunge's laboratory, and published in Hoppe and Seyler's *Zeitschrift für Physiologische Chemie;* at the start it is natural to assume that the milk of rapidly-growing mammals would be richer in those elements which go especially to produce tissue, namely, albumin and salts. Pröscher's investigations have confirmed this assumption. According to Emil Pfeiffer's analysis, the milk of the human mother is very rich in albumin the first and second day, amounting to 8.6 per cent. After eight to fourteen days the albuminous element sinks to 2.5 per cent. It then remains at this point during the first three months, to fall during the second half year to 1.6 per cent. According to this the growth of the infant is greatest during the first eight weeks; it reaches its climax of increase in the third to the fifth week. With the decline in the albuminous element the daily increase in weight sinks, and averages in the middle of the first year daily only 0.39 per cent. of the entire weight.

As in man, so the element of albumin influences the rapidity of growth in all the mammals. In the following list the milk of the various animals is named according to the proportion of albuminous matter which it contains, beginning with the lowest: human milk, 8.6 per cent., she-ass, mare, goat, elephant, camel, cow, buffalo, swine, sheep, dolphin, dog, cat, reindeer.

A characteristic difference in the constituency of the milk is conditioned by the climate. The milk of animals living in the south is poor in fat, but rich in sugar—*e. g.*, the ass's, camel's, horse's, and elephant's. The reverse is true of animals living in the north, as reindeer's milk. This corresponds also to observations made in regard to the various human tribes. Those living in the south live principally upon strong, saccharine foods, while the inhabiants of the north much prefer fatty food. Strange to say, elephant's milk is an exception ; its milk is five times richer in fatty materials than cow's milk (20.5 : 4.5 per cent.). The ideal milk for the creamery would be dolphin's milk, which contains 43.7 per cent. of fat. The exception in the case of the elephant I account for by the fact that this animal was originally a northern animal, inhabited a colder climate, but, in spite of the fact that it has migrated to a warmer climate, still retains the same constituency in its milk. In like manner, from the richness of human milk in sugar and its poverty in fat, we may conclude with the same probability that man lived originally in a warm climate.—*Idem.*

